# Bile acid interactions with neurotransmitter transporters

**DOI:** 10.3389/fncel.2023.1161930

**Published:** 2023-04-26

**Authors:** Tiziana Romanazzi, Daniele Zanella, Manan Bhatt, Angela Di Iacovo, Aurelio Galli, Elena Bossi

**Affiliations:** ^1^Laboratory of Cellular and Molecular Physiology, Department of Biotechnology and Life Sciences, University of Insubria, Varese, Italy; ^2^Ph.D. School in Experimental and Translational Medicine, University of Insubria, Varese, Italy; ^3^Department of Surgery, University of Alabama at Birmingham, Birmingham, AL, United States; ^4^Center for Research in Neuroscience, University of Insubria, Varese, Italy

**Keywords:** SLC6, bile acids, dopamine transporter, obeticholic acid (OCA), two-electrode voltage clamp, GABA transporter 1, glycine transporter

## Abstract

Synthesized in the liver from cholesterol, the bile acids (BAs) primary role is emulsifying fats to facilitate their absorption. BAs can cross the blood-brain barrier (BBB) and be synthesized in the brain. Recent evidence suggests a role for BAs in the gut-brain signaling by modulating the activity of various neuronal receptors and transporters, including the dopamine transporter (DAT). In this study, we investigated the effects of BAs and their relationship with substrates in three transporters of the solute carrier 6 family. The exposure to obeticholic acid (OCA), a semi-synthetic BA, elicits an inward current (I_BA_) in the DAT, the GABA transporter 1 (GAT1), and the glycine transporter 1 (GlyT1b); this current is proportional to the current generated by the substrate, respective to the transporter. Interestingly, a second consecutive OCA application to the transporter fails to elicit a response. The full displacement of BAs from the transporter occurs only after exposure to a saturating concentration of a substrate. In DAT, perfusion of secondary substrates norepinephrine (NE) and serotonin (5-HT) results in a second OCA current, decreased in amplitude and proportional to their affinity. Moreover, co-application of 5-HT or NE with OCA in DAT, and GABA with OCA in GAT1, did not alter the apparent affinity or the I_max_, similar to what was previously reported in DAT in the presence of DA and OCA. The findings support the previous molecular model that suggested the ability of BAs to lock the transporter in an occluded conformation. The physiological significance is that it could possibly avoid the accumulation of small depolarizations in the cells expressing the neurotransmitter transporter. This achieves better transport efficiency in the presence of a saturating concentration of the neurotransmitter and enhances the action of the neurotransmitter on their receptors when they are present at reduced concentrations due to decreased availability of transporters.

## 1. Introduction

Bile acids (BAs) are a large family of molecules which act as a detergent supporting absorption and digestion of lipids. They are derived from cholesterol, synthesized in the liver, and accumulated in the gallbladder. In response to food intake, BAs are released into the duodenum (López et al., 2017). The enterohepatic circulation allows their reabsorption, and through the portal vein, they return to the liver. In this process, a small pool of BAs enter the systemic circulation.

Under physiological conditions, 20 different kinds of BAs can be detected in rodents and human brains (Mano et al., 2004; Higashi et al., 2017; Pan et al., 2017). Both conjugated and unconjugated BAs pass the blood-brain barrier (BBB) to reach the brain. Unconjugated BAs diffuse through the BBB, whereas conjugated BAs need a specific transporter to be translocated, namely the organic anion transporting polypeptides (OATP) (Lee et al., 2005), the organic anion transporter (OAT) (Kikuchi et al., 2003; Roberts et al., 2008) and the apical sodium-dependent bile acid transporter (ASBT) (McMillin et al., 2015). BAs can also be directly synthesized in the brain from cholesterol by cytochrome P450 46A1 (CYP46A1), an enzyme expressed only in neurons (Kiriyama and Nochi, 2019).

It is well-established that BAs influence complex physiological behaviors through their targets in the brain. Both the Takeda G protein-coupled receptor 5 (TGR5, also GPBAR1) (Maruyama et al., 2002) and the nuclear farnesoid X receptor (FXR) (Makishima et al., 1999; Wang et al., 1999) are expressed in the brain. TGR5 was found in cultured astrocytes and neurons. Its stimulation activated the TGR5-adenylate cyclase (Keitel et al., 2010), or acted as anorexigenic protein controlling satiety in response to physiological feeding (Perino et al., 2021). FXR is localized in the nucleus of the brain cortex and hippocampal neurons (Huang et al., 2016). In FXR knockout mice, changes in neurotransmitter homeostasis, such as glutamate, γ-aminobutyric acid (GABA), serotonin (5-HT), and norepinephrine (NE), were detected, suggesting that FXR activity mediates the regulation of neurotransmitters (Schmidt et al., 2015).

There is also the possibility of a physiological role for BAs in the modulation of brain activity through pathways other than their physiological targets. Direct activation of multiple targets important in neuromodulation has been described, such as muscarinic receptors (Raufman et al., 2002), NMDA (Schubring et al., 2012) and GABA receptors (Yanovsky et al., 2012) and some ion channels (Wiemuth et al., 2014; Kiriyama and Nochi, 2019). This aspect of BAs is, however, underrepresented in the field.

Recently, we have shown that OCA (a semi-synthetic BA analog with promising therapeutic applications) and the dopamine transporter DAT (SLC6A3) interact directly (Romanazzi et al., 2021). This was demonstrated through heterologous expression in *Xenopus laevis* oocytes and two-electrode voltage clamp experiments investigating the currents under different conditions. Using molecular docking simulations, we also identified putative OCA binding sites, which could alter the transport cycle stabilizing DAT in an occluded conformation.

Dopamine transporter is a neurotransmitter sodium symporter (NSS) belonging to the solute carrier 6 (SLC6) family. The SLC6 transporter family is essential in the homeostasis of neurotransmitters, nutrient molecules and, osmolytes; it has a fundamental role in controlling membrane potential (Sonders et al., 1997; Niello et al., 2020) in both central and peripheral nervous systems (Pramod et al., 2013). DAT is a member of the monoamine transporter (MAT) subfamily, together with serotonin (SLC6A4) and norepinephrine (SLC6A2) transporters, with which it shares significant sequence homology. It is also closely related to GAT1 (GABA Transporter SLC6A1) and glycine transporters (SLC6A5 and SLC6A9), which belong to the same neurotransmitter and amino acid transporter family (Castagna et al., 2022) and regulate, with other transporters of the family, the homeostasis of the two inhibitory neurotransmitters. These two transporters have been well characterized and studied for their electrical properties by our group (Pérez-Siles et al., 2012; Bhatt et al., 2023). Furthermore, they elicit larger transport currents than DAT when expressed in *Xenopus laevis* oocytes. In this work, we investigated the interaction of BAs with DAT, and assessed if those interactions are shared with other SLC6 family members, namely GAT1 and GlyT1b.

## 2. Materials and methods

### 2.1. Solutions

The ND96 and NDE solutions used during oocyte preparation and culture had the following compositions (in mM): ND96: NaCl 96, KCl 2, CaCl_2_ 1.8, MgCl_2_ 1, HEPES 5, pH7.6; NDE: ND96 plus 2.5 mM pyruvate, 50 μg/ml gentamycin sulfate, penicillin streptomycin solution, 10 U/ml. The external control solution for electrophysiological studies (ND98) had the following compositions (in mM): NaCl 98, MgCl_2_ 1, CaCl_2_ 1.8 and HEPES 5. The final pH was adjusted to 7.6 with NaOH. Substrates used were dopamine (DA) (Merck, Italy), γ-Aminobutyric acid (GABA) (Merck, Italy), SKF89976A (Merck, Italy), glycine (Merck, Italy), 5-hydroxytryptamine (5-HT) (Merck, Italy), lithocholic acid (LCA) (Merck, Italy), and obeticholic acid (OCA) (Adipogen, Füllinsdorf, Switzerland). LCA and OCA powder were dissolved in DMSO at 50 and 100 mM, respectively.

### 2.2. Oocytes collection and cRNA preparation

The oocytes were obtained from adult *Xenopus laevis* females. Animals were anesthetized in 0.1% (w/v) MS222 (tricaine methanesulfonate; Merck, Italy) solution in water. Abdomens were sterilized with the antiseptic agent (Povidone-iodine 0.8%), laparotomy was performed, and portions of the ovary were collected. The oocytes were treated with 1.5 mg/ml collagenase (collagenase type IA from *Clostridium histolyticum*, C0130 from Merck, Italy) in ND96 calcium-free for at least an hour at 18°C. Healthy and fully grown oocytes were selected and stored at 18°C in NDE solution (Bhatt et al., 2022). The experimental protocol was approved locally by the Committee of the “Organismo Preposto al Benessere Animale” of the University of Insubria, Varese, Italy, and nationally by Ministero della Salute (n. 449/2021-PR), Italy. cDNAs were linearized with restriction enzymes, *in vitro* capped and transcribed using 200 units of T7 RNA polymerase. All enzymes were supplied by Promega (Italy). For specifications about the constructs, see Table 1. The day after the removal, the oocytes were injected with cRNA using a manual microinjection system (Drummond Scientific Company, Broomall, PA, USA). Injected concentrations were 12.5 ng/50 nl for mDAT, rGAT1, and 25 ng/50 nl for rGlyT1b. Then, the oocytes were incubated at 18°C for 2–3 days before electrophysiological experiments.

**TABLE 1:** List of transporter gene and vector characteristics, along with molecular methodology used to get the cRNA for oocytes injection.

Transporter	SLC	Organism	GenBank^®^ accession numbers	Vector	Restriction enzyme for linearization
DAT	SLC6A3	*Mus musculus*	AAF85795.1	pGHJ	*Sal*I
GAT	SLC6A1	*Rattus norvegicus*	NP_077347.1	pAMV-PA	*Not*I
GlyT1b	SLC6A9	*Rattus norvegicus*	M88595.1	pRC-CMV	*Cla*I

### 2.3. Electrophysiology

Electrophysiological studies were performed using the two-electrode voltage clamp (TEVC) technique (Oocyte Clamp OC-725; Warner Instruments, Hamden, CT, USA). The controlling software was WinWCP version 4.4.6 (J. Dempster, University of Strathclyde, Glasgow, UK) or Clampex (Molecular Devices, Sunnyvale, CA, USA). Borosilicate microelectrodes, with a tip resistance of 0.5–4 MΩ, were filled with 3 M KCl. Bath electrodes were connected to the experimental oocyte chamber via agar bridges (3% agar in 3 M KCl). The holding potential was kept at −60 mV for all the experiments. Data analysis was performed using Clampfit 10.2 software (Molecular Devices, Sunnyvale, CA, USA); OriginPro 8.0 (OriginLab Corp., Northampton, MA, USA) and GraphPad Prism 8.0.2 (GraphPad Software, Boston, MA, USA) were used for statistical analysis and figure preparation. The values reported are the mean ± SE; statistical analysis was done with ANOVA one-way and repeated measures ANOVA.

We have defined I^0^_OCA_ or I^0^_LCA_ as the transient current elicited due to the first (0) exposure of OCA or LCA, respectively. I’_OCA_ refers to the transient current elicited by any other exposure to OCA followed by the first one. I*_*S*_* (*S* is substrate) represents the current elicited by the transport of the indicated substrate.

## 3. Results

### 3.1. Dopamine and OCA interaction

**FIGURE 1:**
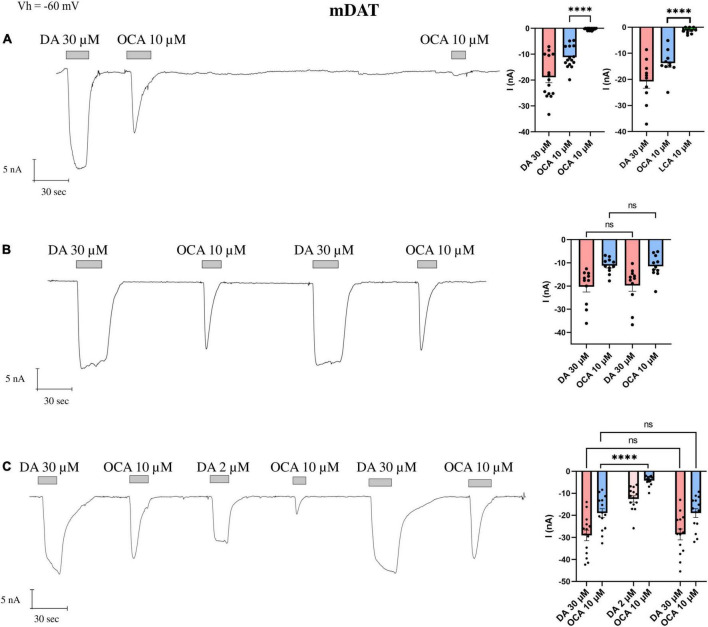
The displacement of OCA from mDAT by DA. Representative traces of currents recorded by TEVC from oocytes expressing mDAT exposed to OCA (left) and mean of the indicated currents (right). **(A)** Currents after the first and second exposure to OCA, with an interval of 5′ wash with ND98 alone; mean current (nA) ± SE, from *n* = 15 oocytes, *n* = 4 batches; I^0^_OCA_ vs. I’_OCA_ on the same oocyte: one-way ANOVA *F*_(2,42)_ = 18.15, ^****^p < 0.0001 followed by Bonferroni’s multiple comparison test; I^0^_OCA_ vs. I’_*LCA*_ same oocyte: ^****^p < 0.0001. **(B)** Currents after the first and second exposure to OCA with washing ND98 and ND98 plus DA 30 μM after the first OCA exposure; mean current (nA) ± SE of 11/3 n/N; repeated measure one-way ANOVA *F*_(1.220, 12.20)_ = 20.71, *p* = 0.0001 between columns and *F*_(10, 30)_ = 9.253, ^****^p < 0.0001 between rows, followed by Bonferroni’s multiple comparison test. I_DA_ vs. I’_DA_: *p* = 0.8737 and I^0^_OCA_ vs. I’_OCA_: *p* > 0.9999. **(C)** Currents after the first and second exposure to OCA with washing ND98 and ND98 plus DA 2 μM after the first OCA exposure; mean current (nA) ± SE of 13/2 n/N; repeated measure one-way ANOVA *F*_(2.838, 36.89)_ = 62.26, ^****^p < 0.0001 between columns and *F*_(13, 65)_ = 10.44, ^****^p < 0.0001 between rows followed by Bonferroni’s multiple comparison test. I_DA_ vs. I’_DA_: *p* > 0.9999; I^0^_OCA_ vs. I”_OCA_: *p* > 0.9999; I^0^_OCA_ vs. I‘_OCA_ after DA 2 μM: ^****^p < 0.0001.

The perfusion of DA (30 μM), over a *X. laevis* oocyte heterologously expressing mDAT, generated an inward transport current (I_DA_) of about 25 nA, and OCA 10 μM elicited an inward transient current (I^0^_OCA_) of about 60% of I_DA_; a second applications of OCA (I’_OCA_) did not elicit a similar response (Figure 1A). The amplitude of I^0^_OCA_ and I’_OCA_ (nearly undetectable) measured on the same oocytes are statistically different (*p* < 0.0001). Their relationship is independent of the washing time (from 1 to 15 min) or the concentration of the second dose (data not shown). Furthermore, repeated applications of another BA with lower affinity (LCA, Figure 1A histogram on the right) were not able to generate a current as well. These data support the hypothesis that OCA is still residing in its binding sites.

Next, we tested whether the application of a substrate was able to restore I^0^_OCA_. After the first exposure to OCA, cells were washed with ND98, followed by DA 30 μM perfusion. The second exposure to the OCA elicited a current I’_OCA_ with an amplitude identical to I^0^_OCA_ (Figure 1B). The amplitude of I’_DA_ did not significantly differ from the first I_DA_.

To investigate the dependency of the amplitude of the I’_OCA_ on DA doses, we tested the concentration of DA 2 μM, proximal to the K_0.5_ for mDAT (Sonders et al., 1997; Romanazzi et al., 2021), after the first exposure to OCA. The second application of OCA 10 μM elicited a detectable I’_OCA_, but with a significantly reduced amplitude (40% of I^0^_OCA_) (*p* < 0.0001).

Finally, a third application of OCA 10 μM following the ND98 plus DA 30 μM elicited an I’_OCA_ identical in amplitude (*p* > 0.9999) and shape to I^0^_OCA_ (Figure 1C). It is of note that the I^0^_OCA_ is present, independent of previous exposure to DA.

### 3.2. Norepinephrine and serotonin are substrates of mDAT

**FIGURE 2:**
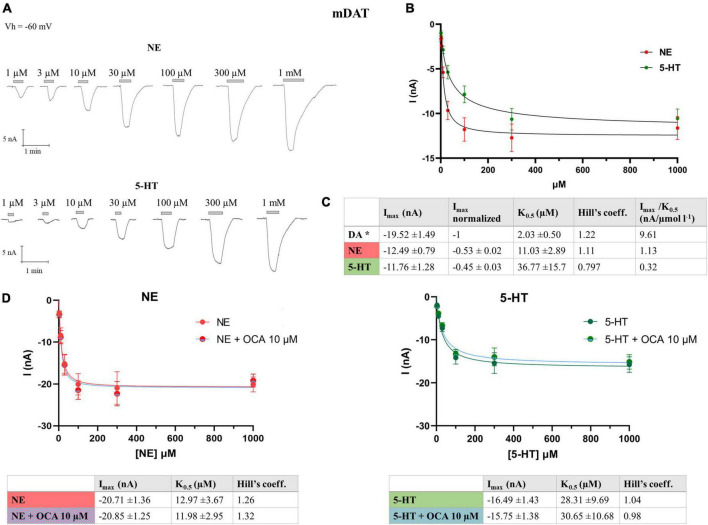
Kinetics parameter of mDAT for NE and 5-HT. **(A)** Representative traces of currents recorded from oocytes expressing mDAT perfused with increasing concentrations of NE, from 1 μM to 1 mM (top) and 5-HT (bottom). **(B)** Data from 14/3 n/N were fitted to Hill’s equation and the data reported in table **(C)**. *Parameter for the DA reported in Romanazzi et al. (2021). **(D)** Data from 11/2 n/N cells perfused with increasing concentrations of NE from 3 μM to 1 mM with or without OCA, 10 μM, were fitted to Hill’s equation and the data reported in table (left) and (right) data from 9/2 n/N cells perfused with increasing concentrations of 5-HT from 3 μM to 1 mM with or without OCA 10 μM were fitted to Hill’s equation and the data reported in the table (NE: I_max_: *p* = 0.9435; K_0.5_: *p* = 0.8341; 5-HT: I_max_: *p* = 0.7259; K_0.5_: *p* = 0.8730).

The pharmacological characterization on *Drosophila melanogaster* dopamine transporter (dDAT) defines the potency of different agonists and antagonists of monoamine transporter (such as fluorexetine, cocaine, norepinephrine, and serotonin) to inhibit the DA radiolabeled uptake (Pörzgen et al., 2001), suggesting that NE and 5-HT could act as DAT substrates. To our knowledge in the literature, there are no clear indications as to the capability of mDAT transporting these two substrates. With this aim, we performed dose-response experiments on *X. laevis* oocytes, heterologously expressing mDAT, in the presence of NE and 5-HT, to record the transport response and define the kinetic parameters. Both substrates were tested at concentrations from 1 μM to 1 mM on the same oocytes, and the mean of the amplitude of the current recorded for each dose was fitted using the Hill’s equation. The value of maximal transport current (I_max_) for NE was −12.49 ± 0.79 nA, and for 5-HT −11.76 ± 1.28 nA, i.e., 53% and 45% of the I_max_ for DA, respectively. The apparent substrate affinity K_0.5_ for NE was 10.76 ± 1.67 μM, and for 5-HT 36.98 ± 10.77 μM (Figure 2). We also calculated the transport efficiency, defined as the ratio of I_max_ and K_0.5_ (I_max_/K_0.5_), of DA, NE, and 5-HT in mDAT (Figure 2C). The substrate preference order was DA > NE > 5-HT, and substrate affinity was also DA > NE > 5-HT.

Moreover, we calculated if the maximal currents and/or the apparent affinity of NE or 5-HT for DAT are affected by OCA. Dose-response experiments were performed in the presence of NE or 5-HT, with and without OCA 10 μM. The current means at the different substrate concentrations were fitted with the Hill’s equation and the resulting kinetic parameters are reported in the table in Figure 2D. These data reveal that OCA does not significantly affect both the affinity and the maximal transport currents for NE and 5-HT, similar to what was previously described for DA (Romanazzi et al., 2021).

### 3.3. Norepinephrine and serotonin are able to displace OCA

**FIGURE 3:**
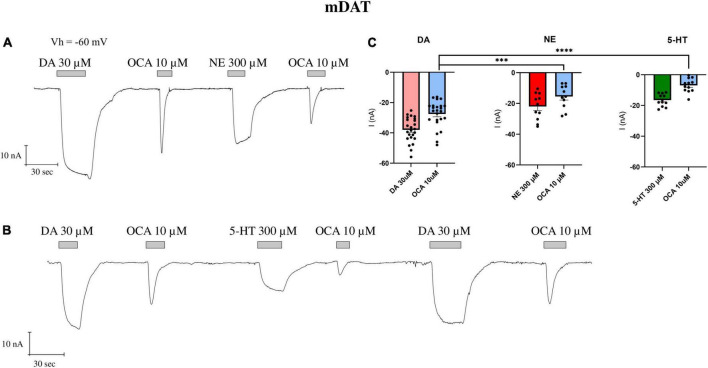
The partial displacement of OCA by NE and 5-HT. **(A)** Representative traces of currents recorded by TEVC (Vh = –60 mV) from oocytes expressing mDAT exposed to NE 300 μM after the first OCA exposure. **(B)** Representative traces of currents recorded by exposure to 5-HT 300 μM after the first OCA perfusion. **(C)** Mean current (nA) ± SE of 11–12/3 n/N; one-way ANOVA *F*_(5,88)_ = 35.51, ^****^p < 0.0001 followed by Bonferroni’s multiple comparison test; I^0^_OCA_ vs. I’_OCA_ after NE: ^***^p = 0.001; I^0^_OCA_ vs. I’_OCA_ after 5-HT: ^****^p < 0.0001.

Since DAT can translocate NE and 5-HT, even with reduced efficiency and lower affinity as reported above, we repeated the experiments of Figure 1, washing the oocytes after the first OCA exposure with ND98 plus NE or 5-HT at saturating concentrations (300 μM). After measuring the amplitude of I_DA_ (DA 30 μM) and I^0^_OCA_ (OCA 10 μM), NE was perfused, which resulted in I_NE_ of about 57% of I_DA_ (as shown above). Interestingly, the following exposure of oocytes to OCA 10 μM elicited an inward transient current I’_OCA,_ which was significantly (*p* = 0.001) reduced in amplitude compared to I^0^_OCA_ (about 56% of I^0^_OCA_) (Figures 3A–C). Applying 5-HT 300 μM, after DA and OCA, generated the I_5–HT_ of about 44% of I_DA_. The subsequent perfusion of OCA 10 μM generated the I’_OCA_, significantly reduced in amplitude (about 25%) than I^0^_OCA_ (*p* < 0.0001) (Figures 3B, C). Finally, after applying NE or 5-HT and the second OCA, if DA was applied at saturating concentrations, OCA elicited a current similar to I^0^_OCA_. These results confirm that only saturating DA can fully displace OCA, moving DAT to the initial cycle conditions (outward facing) and giving rise to the maximal OCA current.

### 3.4. Interaction of BAs with other two members of SLC6 neurotransmitter transporter family, GlyT1b and GAT1

To verify if the interaction of BAs is specific and limited to DAT or extended to other SLC6 transporters, we selected two other members of the family: GAT1 and GlyT1b. The experimental protocol reported in Figure 1 was applied to oocytes expressing rGAT1 or rGlyT1b.

**FIGURE 4:**
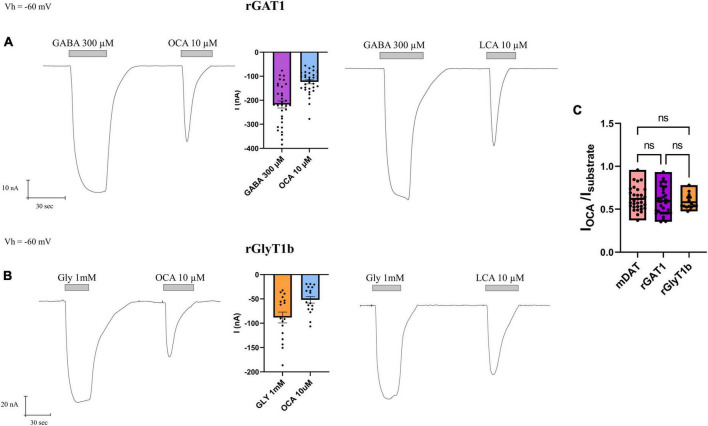
Obeticholic acid (OCA) effect on neurotransmitter transporters GAT1 (SLC6A1) and GlyT1b (SLC6A9). **(A)** Representative traces recorded by TEVC from oocytes expressing rGAT1 exposed to GABA and OCA (left) or GABA and LCA (right); mean of currents (center) (nA) ± SE of 33/6 n/N. **(B)** Representative traces recorded by TEVC from oocytes expressing rGlyT1b exposed to glycine and OCA (left) or glycine and LCA (right); mean of currents (center) (nA) ± SE of 17/4 n/N. **(C)** mean of normalized OCA current for rGlyT1b, mDAT, and rGAT1; (nA) ± SE of 16–34/6 n/N; one-way ANOVA *F*_(2, 79)_ = 0.4982, *p* = 0.6094 followed by Bonferroni’s multiple comparison test. I_OCA_/I_DA_ vs. I_OCA_/I_GABA_: *p* > 0.9999; I_OCA_/I_DA_ vs. I_OCA_/I_Gly_: *p* > 0.9999; I_OCA_/I_Gly_ vs. I_OCA_/I_GABA_: *p* > 0.9999.

The perfusion of GABA 300 μM on oocytes expressing rGAT1 elicited a large and inward transport current I_GABA_ (−217.8 ± 14.57 nA). The subsequent perfusion of OCA (10 μM) generated a proportionally large transient inward I^0^_OCA_ (−123.6 ± 8.32 nA) (Figure 4A).

In oocytes expressing rGlyT1b, the perfusion of glycine 1 mM resulted in an inward transport current I_Gly_ (−88.24 ± 11.15 nA) and OCA 10 μM gave rise to a transient inward I^0^_OCA_ (−51.88 ± 6.70 nA) (Figure 4B).

The perfusion of LCA 10 μM on rGAT1 or rGlyT1b expressing oocytes induced similar inward transient current I_LCA_ (Figures 4A, B), as reported before for mDAT (Romanazzi et al., 2021).

All three transporters investigated here (mDAT, rGAT1, and rGlyT1b) have shown different amplitudes of substrate transport currents (∼25, ∼200, and ∼100 nA, respectively). In the presence of OCA (or LCA), the amplitude of the I^0^_OCA_ (or I^0^_LCA_) was about 60% of the substrate currents (I_*S*_), as shown in Figure 4C. There were no significant differences between the normalized OCA currents induced in the different transporters considered (*p* > 0.99), indicating that OCA has comparable effects in all transporters tested, effects that are proportional to their specific transport-associated current amplitude.

### 3.5. OCA acts similarly in mDAT and rGAT1

**FIGURE 5:**
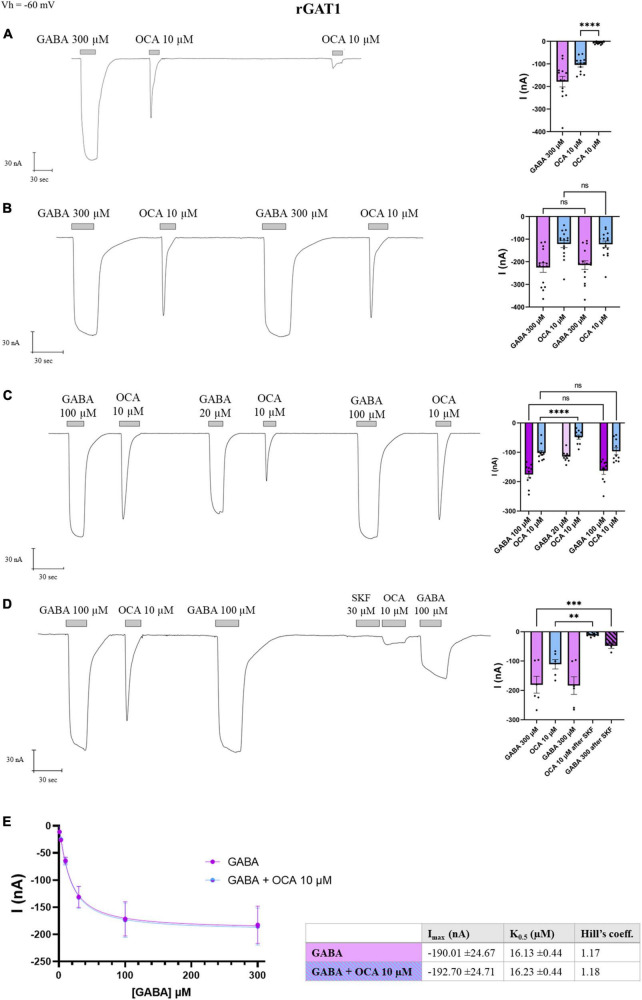
Obeticholic acid (OCA) relationship with rGAT1. Representative traces of currents recorded by TEVC from oocytes expressing rGAT1 exposed to OCA (left) and mean of the indicated currents (right). **(A)** Currents after the first and second exposure to OCA with an interval of 5′ wash with ND98 alone; mean current (nA) ± SE of 13/3 n/N; one-way ANOVA *F*_(2,36)_ = 35.68, ^****^p < 0.0001 followed by Bonferroni’s multiple comparison test: I^0^_OCA_ vs. I’_OCA_: ^****^p < 0.0001. **(B)** Currents after the first and second exposure to OCA with washing ND98 and ND98 plus GABA 300 μM after the first OCA exposure; mean current (nA) ± SE of 15/3 n/N; repeated measure one-way ANOVA *F*_(1.246, 17.44)_ = 45.80, ^****^p < 0.0001 between columns and *F*_(14, 42)_ = 14.93, ^****^p < 0.0001 between rows followed by Bonferroni’s multiple comparison test. I_GABA_ vs. I’_GABA_: *p* = 0.1599 and I^0^_OCA_ vs. I’_OCA_: *p* > 0.9999. **(C)** Currents after the first and second exposure to OCA with washing ND98 and ND98 plus GABA 20 μM after the first OCA exposure; mean current (nA) ± SE of 10/2 n/N; repeated measure one-way ANOVA *F*_(1.765, 15.89)_ = 38.97, ^****^p < 0.0001 between columns and *F*_(9, 45)_ = 10.44, ^****^p < 0.0001 between rows followed by Bonferroni’s multiple comparison test. I_GABA_ 100 μM vs. I’_GABA_ 100 μM: *p* > 0.4177; I^0^_OCA_ vs. I’_OCA_ after GABA 100 μM: *p* > 0.9999; I^0^_OCA_ vs. I’_OCA_ after GABA 20 μM: ^****^p < 0.0001. **(D)** Current after SKF 30 μM perfusion, followed by the second OCA; mean current nA ± SE of 6/2 n/N; one-way ANOVA *F*_(3,18)_ = 17.02, ^****^p < 0.0001 followed by Bonferroni’s multiple comparison test: I^0^_OCA_ vs. I’_OCA_ after SKF: *p* = 0.0019; I_GABA_ vs. I’_GABA_ after SFK: *p* = 0.0003. **(E)** Data from 8/1 n/N cells perfused with increasing concentrations of GABA from 3 to 300 μM with or without OCA 10 μM were fitted to Hill’s equation and the data reported in the table (I_max_: *p* = 0.9523; K_0.5_: *p* = 0.8170). ^**^p < 0.01, ^***^p < 0.001.

Taking advantage of the relatively larger transport-associated current generated by GAT1, we tried to measure the amplitude of I’_OCA_, expecting that in GAT1 the second exposure of OCA might result in a detectable current. The I’_OCA_ indeed generated a current; however, significantly reduced to 6% of I^0^_OCA_ (−6.64 ± 1.47 nA, *p* < 0.0001) (Figure 5A).

After GABA-OCA application, perfusion of GABA 300 μM elicited I_GABA_ with an amplitude comparable to the first one (*p* = 0.1599), and the re-exposure to OCA 10 μM induced an inward transient current of I^0^_OCA_ (*p* > 0.99) (Figure 5B), consistent with the observation for DAT (Figure 1B).

Moreover, if after the first GABA-OCA application, the oocyte received GABA 20 μM [concentration proximal to rGAT1 K_0.5_ at −60 mV (Fesce et al., 2002)], the I’_OCA_ was reduced to the 53% of the I^0^_OCA_ (*p* < 0.0001) (Figure 5C). Exposing the transporter again to GABA at saturating concentrations, the amplitude of I’_OCA_ was not statistically different from I^0^_OCA_ (*p* > 0.9999). Furthermore, the application of OCA did not alter the amplitude of I_GABA_ (Figure 5C). SKF89976A is a specific GAT1 blocker that abolishes the pre-steady-state currents completely and the transport-associated currents partially (Mager et al., 1993; Cherubino et al., 2012). After GABA-OCA-GABA application, the oocyte was exposed for 30 s to SKF89976A 30 μM and followed by the second exposure to OCA that lead to a significant reduction of I’_OCA_ (10% of the I^0^_OCA_) (*p* = 0.0019). This reduction was consistent with the decrease of the GABA transport current (*p* = 0.0003) (Figure 5D). The fact that in the presence of the GAT1-specific inhibitor (SKF-89976A), the OCA-induced current was drastically reduced, supports that the OCA current is directly mediated via rGAT1.

This data reported in Figure 4 indicates that OCA interacts directly with GAT1, in the absence of GABA. The effect of OCA on the kinetic parameters of GAT1 was also investigated. Currents generated from increasing concentrations of GABA were unaltered by the presence of OCA 10 μM (Figure 5E). Current means were fitted with the Hill’s equation and kinetic parameters are reported in the table in Figure 5E. OCA does not significantly affect either the affinity or the maximal transport currents of rGAT1, similar to what was previously described for mDAT (Romanazzi et al., 2021).

## 4. Discussion and conclusion

Bile acids are amphipathic molecules synthesized in the liver from cholesterol and released into the duodenum. Their physiological role as fat absorption adjuvants and signaling molecules involved in metabolism is well-established. Recently, a possible role as a direct modulator of brain function has emerged (Raufman et al., 2002; Schubring et al., 2012; Yanovsky et al., 2012). In our previous work, we showed that in *Xenopus laevis* oocytes expressing mDAT, OCA and LCA elicited a sodium transient inward current (Romanazzi et al., 2021). The molecular docking simulations identified multiple putative binding sites for OCA. In our interpretation, upon binding OCA induced conformational changes. The rearrangement of the transmembrane domains in positioning in the occluded conformation, opens a temporary ion conductance. In this work, we examined the interaction of BAs with DAT, GAT1, and GlyT1b, all neurotransmitter transporters of the SLC6 family, to study the specificity of the binding and the mechanics involved.

As reported, OCA can bind and occlude DAT, but the effectiveness of the occlusion is debatable (Romanazzi et al., 2021). To elucidate this phenomenon, we tested the displacement of bound OCA, investigating whether the transporter gets blocked, or if OCA locks the transporter until a substrate with stronger affinity induces reopening of it. As shown in Figure 1, the second application of OCA does not elicit any currents (I’_OCA_) in DAT; a regular transient inward current is visible only when preceded by the perfusion of DA. When the oocytes are perfused with a concentration of DA lower than the saturating one, I’_OCA_ is significantly smaller than I^0^_OCA_. These observations suggest that the OCA molecules bound to DAT are not displaced from the transporter by simple buffer washing, and that only the presence of saturating concentrations of DA completely remove OCA from its binding site. This results in a second OCA current with reduced amplitude (Figure 1C).

The transporter becomes able to respond again to OCA only when it completes a transport cycle, translocating the substrate inside the cell and returning in an outward facing conformation. The amplitude of I’_OCA_ is similar to I^0^_OCA_ only when all the transporters expressed reach the condition of maximal activity (I_max_). We thus hypothesize that I’_OCA_ amplitude is directly correlated to the unbinding of the OCA from the transporter. To test this hypothesis, we exposed the oocytes to lower affinity substrates of DAT, like NE and 5-HT. As our data show (Figure 2), these are transported with lower efficiency without reaching the I_max_ values recorded for DA. These substrates should fail to fully remove OCA from DAT, thereby yielding lower I’_OCA_. Indeed, the perfusion of one of these substrates at its saturating concentration after the first OCA exposure results in a reduced amplitude of I’_OCA_ (Figures 3A, B), similar to the one resulting in the presence of DA at concentrations close to the K_0.5_.

Observing the recovery of the amplitude of I’_OCA_ after the substrate perfusion, the displacement of OCA by saturating DA, NE, or 5-HT is consistent with their respective K_0.5_ for DAT. Our interpretation is that the lower binding energy of the secondary substrates NE and 5-HT to the substrate binding site might not be enough to drive all the transporters to complete the transport cycle, as suggested by the lower I_max_.

While the interaction of OCA with DAT is interesting, the larger question is whether it is limited to DAT. Therefore, we assessed the effect of OCA on other members of the SLC6 family involved in neurotransmission, namely GAT1 (member of the GABA transporters subfamily) and GlyT1b (neurotransmitter member of the amino acid transporters). In oocytes expressing rGlyT1b or rGAT1, the perfusion of BAs produces a current that is larger than that recorded in mDAT, and proportional to the current induced by the specific substrate. The fact that the OCA current was proportional to the transport current supports the idea that this conductance triggered by OCA should be mediated by the rearrangement of the transporter (Figures 4, 5).

This binding is not exclusive to OCA perfusion, as GlyT1b and GAT1 respond to the application of LCA as well. LCA is a natural bile acid that shares with OCA the same sterol-based structure, with the ethyl and hydroxyl groups present in OCA substituted by hydrogen in LCA. This observation is important not only to assess that a similar interaction with transporters occurs even in the presence of different residues, sharing common binding mechanisms, but also concerning the pathophysiological role of endogenous BAs, which, for example, increase in the systemic circulation after bariatric surgery and may act on different targets in the central nervous and enteric systems (Mano et al., 2004).

We further investigated these interactions, using the same approach of multiple OCA applications, to characterize the removal of OCA bound to these transporters. In GlyT1b and GAT1, the second exposure to BAs does not elicit reliable responses, suggesting that as in DAT, the first BA bound to the transporter makes its binding site inaccessible to other BAs molecules. BAs do not modify GAT1 kinetic transport parameters, confirming that these molecules act only on the transporters in the absence of substrate. Perfusion of GABA at a concentration lower than saturating, after the first OCA application, caused an I’_OCA_ current reduced in amplitude, confirming what was previously seen in DAT.

**FIGURE 6:**
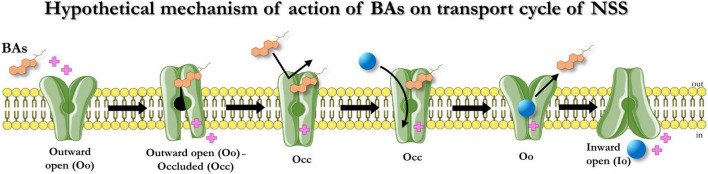
Hypothetical mechanism of action of BAs on the transport cycle of NSS. In presence of BAs, the BA molecule binds the transporters (Oo), opens a transient conductance (Oo-Occ), and then freezes the protein in an occluded conformation (Occ) that makes it resistant to other BAs-mediated alteration of membrane potential. The occluded state can be recovered only in the presence of the physiological substrates that allow the transporter to switch from Oo to Io. The binding of the physiological transport induces the unbinding of OCA from the protein.

In conclusion, BAs act on the tested members of the SLC6 family members similarly: upon binding, they induce a conformation rearrangement that opens a transient conductance and while bound, the BAs prevent the action of another BA molecule. This occluded conformation can be recovered only in the presence of substrates (Figure 6). Consequently, the BAs do not act on the kinetic parameters of the transport. A possible physiological significance of this behavior is avoiding the summation of small depolarizations in the cells expressing the neurotransmitter transporters, permitting better transport efficiency in the presence of saturating concentrations of neurotransmitter, and enhancing the action of neurotransmitters on their receptors when they are present at reduced concentrations due to decreased availability of transporters, partially blocked in the occluded conformation by the Bas. Also, by avoiding the summation of small inward (depolarizing) currents elicited by the Bas, the cell could prevent the alteration of neuronal excitability or the efficiency controls of the neurotransmitter homeostasis by the uptake by astrocytes (Pramod et al., 2013). The transporter-occluded state is recovered when the transporter works at maximal efficiency with a saturating concentration of substrate.

The characterization of the action of BAs on three members of SLC6 is a first step in investigating the relationship between the SLC protein and BAs, a class of molecules that are also present in the brain. Identifying the structural determinants of these transporters involved in BAs binding might provide fundamental information in drug discovery to identify new therapeutic uses for BAs, considering that studies focused on the use of BAs as a treatment for brain conditions are rapidly growing (Monteiro-Cardoso et al., 2017; Kiriyama and Nochi, 2019; Grant and Demorrow, 2020) and that multiple studies have already proven their value as signaling molecules (Reddy et al., 2018; Perino et al., 2021) in the gut-brain relationship.

## Data availability statement

The raw data supporting the conclusions of this article will be made available by the authors, without undue reservation.

## Ethics statement

This animal study was reviewed and approved by the Committee of the “Organismo Preposto al Benessere degli Animali” of the University of Insubria and nationally by Ministero della Salute (permit nr. 449/2021-PR).

## Author contributions

TR, MB, and AD performed the experiments. TR analyzed the data and prepared the figures. TR and EB wrote the manuscript. DZ and MB contributed to editing the manuscript. AG, DZ, and EB designed and supervised the studies. All authors contributed to the article and approved the submitted version.
